# The association of chronic anxiousness with cardiovascular disease and mortality in the community: results from the Gutenberg Health Study

**DOI:** 10.1038/s41598-020-69427-8

**Published:** 2020-07-24

**Authors:** Iris C. Reiner, Ana N. Tibubos, Antonia M. Werner, Mareike Ernst, Elmar Brähler, Jörg Wiltink, Matthias Michal, Andreas Schulz, Philipp S. Wild, Thomas Münzel, Natalie Arnold, Seyed Hamidreza Mahmoudpour, Karl J. Lackner, Norbert Pfeiffer, Manfred E. Beutel

**Affiliations:** 1 Department of Psychosomatic Medicine and Psychotherapy, University Medical Center of the Johannes Gutenberg-University Mainz, Untere Zahlbacher Str. 8, 55131 Mainz, Germany; 2 Preventive Cardiology and Preventive Medicine-Center for Cardiology, University Medical Center of the Johannes Gutenberg-University Mainz, Langenbeckstr. 1, 55131 Mainz, Germany; 3 Center for Cardiology-Cardiology I, University Medical Center of the Johannes Gutenberg-University Mainz, Langenbeckstr. 1, 55131 Mainz, Germany; 4 Institute of Medical Biostatistics, Epidemiology and Informatics, University Medical Center of the Johannes Gutenberg-University Mainz, Obere Zahlbacher Str. 69, 55131 Mainz, Germany; 5 Institute of Clinical Chemistry and Laboratory Medicine, University Medical Center of the Johannes Gutenberg-University Mainz, Langenbeckstr. 1, 55131 Mainz, Germany; 6 Department of Ophthalmology, University Medical Center Mainz of the Johannes Gutenberg-University Mainz, Mainz, Germany; 7 Center for Thrombosis and Hemostasis (CTH), University Medical Center of the Johannes Gutenberg-University Mainz, Langenbeckstr. 1, 55131 Mainz, Germany; 8 German Center for Cardiovascular Research (DZHK), Partner Site Rhine-Main, Langenbeckstr. 1, 55131 Mainz, Germany; 9 Center for Translational Vascular Biology (CTVB), University Medical Center of the Johannes Gutenberg-University Mainz, Langenbeckstr. 1, 55131 Mainz, Germany

**Keywords:** Psychology, Cardiology, Risk factors

## Abstract

In a large German community sample of adults, we investigated the association of chronic anxiousness with cardiovascular disease and mortality. Self-reported anxiousness from 11,643 German adults between 40 and 80 years of age from the Gutenberg Health Study (GHS) was analyzed over 5 years. Multivariable regression modeling assessed the relation between the variables, cardiovascular disease and mortality. Twelve percent of the participants reported consistently raised (chronic) anxiousness over at least 2.5 years. Anxiousness was more often reported by female, younger participants with a lower socioeconomic status, smokers and those with a family history of stroke and myocardial infarction. New onset of cardiovascular disease was linked to chronic anxiousness in men and new onset of anxiousness in women. However, chronic anxiousness did not predict all-cause mortality. Our results revealed that anxiousness is highly prevalent in German adults from middle to old age, affecting women in particular. In our study, we found sex-specific associations between new onset of cardiovascular disease and different forms of anxiousness in men and women. We suggest that even subclinical levels of anxiety need to be considered as cardiovascular risk factors. To elucidate potential harm of anxiousness for mental and physical health, we propose sex-specific analyses in further research studies, taking age and the course of anxiousness into account.

## Introduction

Anxiety disorders are among the most frequent mental disorders in the German population with a 12-month prevalence of more than 14%^1^. The prevalence of anxiety disorders in cardiovascular and other somatic diseases was even higher, estimated at 30% or more^2^. Among different types of anxiety disorders, general anxiety disorder (GAD) has been found to be most strongly associated with cardiovascular disease (CVD)^3^. Life-threatening somatic illness may induce anxiety, and the presence of comorbid anxiety disorders with serious medical disease has carried increased risks for disability and medical prognosis^4–6^.

Several meta-analyses indicated that anxiety disorders might also be an independent risk factor in the development of CVD^7–10^. Anxiety disorders have been associated with an excess risk of about 26% for the development of coronary artery disease in healthy participants^8^. For instance, Smeijers et al.^11^ found that high levels of anxiety and anger symptoms immediately preceding myocardial infarction (MI) were risk factors for long-term mortality in older patients. In a meta-analysis of 46 cohort studies^10^, anxiety was associated with a 41% higher risk of cardiovascular mortality and coronary heart disease, a 71% higher risk of stroke, and a 35% higher risk of heart failure. An increasing number of studies have discussed the impact of clinically relevant anxiety not only on CVD related mortality, but also on all cause-mortality^12–15^. By contrast, Miloyan et al.^16^ found no increase in overall mortality in anxiety disorders (based on 36 cohort studies) after adjusting for publication bias. Yet, biological and behavioral underpinnings of the association of anxiety disorders, medical illness and mortality are not fully understood.

Chronic anxiety disorders foster additional mental disorders, for example depression, further anxiety disorders, sleep disturbances, somatoform, pain, addiction (alcohol, sedatives) and eating disorders and involve an increased risk for suicide^17^. The high utilization of medical care has been portrayed by studies from primary care: Anxiety, depressive and somatoform disorders affected about 40% of the patients (point prevalence) in German general practices; anxiety disorders affected 15.7% (specific phobia included) and most of them overlapped with depressive and somatoform disorders^18^.

Characterized by somatic tension and hyperarousal, anxiousness is affecting substantial proportions of clinical and nonclinical populations. This frequently includes uncontrollable worry about several areas of life, the hallmark of generalized anxiety disorder^19^. Anxiousness might be expected in life-transitions or situations of uncertainty, such as serious somatic illness. Several studies have indicated that not only clinically diagnosed anxiety disorders, but also subclinical anxiousness may be related to cognitive, emotional and physical impairments^20–22^.

While CVD and mortality have been associated to different forms of anxiety disorders, less is known about chronic subclinical anxiousness in the general population, its components and potential associations to CVD and life expectancy. Relationships between anxiety disorders and somatic diseases are quite complex. According to Celano et al.^23^, the association between anxiety disorders and cardiac outcomes is mediated by unhealthy diet (cholesterol, total calorie intake, sedentary life style), decreased physical activity and lack of compliance with medical recommendations such as smoking cessation. Physiological pathways such as increased inflammation, ventricular, platelet and autonomic dysfunction (elevated heart rate) may contribute to cardiovascular risk factors and even mortality. As Tully et al.^3^ have indicated, GAD may have detrimental cardiovascular effects due to its worry component, which has been shown to increase sympathetic and reduce parasympathetic activity. Thus, we expected that particularly chronic anxiousness posed a risk for new onset of cardiovascular disease and increased mortality.

As there are sex differences in the incidence and prevalence, with more women affected by anxiety disorders^24^ and more men with CVD^25,26^, sex-specific associations need to be taken into account investigating the influence of anxiousness on CVD. The present paper aims to address these issues and close some of these research gaps by examining.

- Overall as well as sex-specific associations of chronic anxiousness and new onset of CVD, and
- The predictive value of chronic anxiousness on overall mortality.

## Results

### Anxiousness in a German community sample: frequency and distribution

We investigated a representative community sample with a large age range (40–80 years). Chronic anxiousness was defined with regard to three different time points (see the “Methods” section for details).

Table 1 shows the different chronological forms of anxiousness and their sociodemographic and cardiovascular risk factors as well as associated psychological distress.

**Table 1:** Anxiousness in a German community sample: Sociodemographic features, new onset of cardiovascular disease, cardiovascular risk factors and distress.

Anxiousness	Never	Previous	New onset	Chronic	*p*-value*
	62.1% (*n* = 6,831)	15.8% (*n* = 1741)	10.1% (*n* = 1,108)	12% (*n* = 1,324)	
**Sociodemographics**					
Age (years) (M, SD)	60.2 ± 10.9	58.6 ± 10.6	58.3 ± 10.7	57.6 ± 10.1	< 0.0001
Men (%; n)	57.4 (3,922)	44.5 (774)	44.6 (494)	37.6 (498)	< 0.0001
Socioeconomic status (M, SD)	13.30 ± 4.41	12.90 ± 4.40	13.31 ± 4.28	12.49 ± 4.26	< 0.0001
**Cardiovascular disease and risk factors (%; n)**					
New onset CVD	4.5 (273)	4.3 (66)	5.3 (53)	6.4 (76)	0.0059
Diabetes	10.7 (730)	10.3 (178)	9.0 (99)	8.3 (109)	0.0027
Obesity	25.8 (1759)	24.6 (428)	25.9 (287)	26.4 (349)	0.76
Smoking	13.5 (921)	15.5 (270)	17.2 (190)	18.1 (239)	< 0.0001
Hypertension	55.7 (3,792)	51.1 (888)	49.7 (550)	50.7 (671)	< 0.0001
Dyslipidemia	34.3 (2,335)	33.9 (588)	32.5 (559)	36.3 (487)	0.71
FH of MI/Stroke	21.8 (1,488)	25.8 (449)	24.8 (428)	26.7 (360)	< 0.0001
**Distress (%; n)**					
Social Phobia	1.3 (90)	3.4 (59)	6.7 (74)	19.4 (254)	< 0.0001
Panic attack (past 4 weeks)	1.0 (70)	4.5 (78)	7.3 (80)	20.7 (271)	< 0.0001
Sleep disturbance	5.4 (362)	9.9 (168)	14.0 (153)	24.7 (322)	< 0.0001

*N* = 11,004.

FH of MI/Stroke, Family History of Myocardial infarction/Stroke; CVD, Cardiovascular disease sex 1 = male, 0 = female; cardiovascular risk and distress: 0 = does not apply, 1 = applies; *chi^2^ or Kruskal Wallis test; M, means; SD, standard deviations.

A total of 62% of the participants were never, 16% previously anxious, and 10% reported new onset of anxiousness, and 12% chronic anxiousness. Anxiousness was linked to younger age and female sex. New onset of CVD affected 4.5% of those who were never, resp. previously anxious (4.3%). The rate was 5.3% of those with new onset of anxiousness and 6.4% in the chronically anxious. Anxiousness was associated with higher rates of smoking and family history of myocardial infarction/stroke, distress and sleep disturbance as well as lower rates of hypertension. Participants with chronic anxiousness reported social phobia, panic attacks and sleep disturbance most frequently. Sex-specific tables are added as Supplemental material (S1).

### Chronic anxiousness and new onset of CVD

Table 2 presents the regression models of different forms of anxiousness on new onset of CVD, including separate analyses for men and for women.

**Table 2:** Regression model of different chronological forms of anxiousness, sociodemographic as well as cardiovascular risk factors and distress on onset CVD for total sample, men and women.

Variables	Total sample			Men			Women		
	OR	95% CI	*p*-value	OR	95% CI	*p*-value	OR	95% CI	*p*-value
**Form of anxiousness**									
Previous vs never	1.204	0.89–1.61	0.22	1.183	0.78–1.74	0.41	1.185	0.75–1.83	0.45
New onset vs never	1.471	1.05–2.03	0.021	1.344	0.84–2.06	0.19	1.682	1.00–2.72	0.041
Chronic vs never	1.794	1.29–2.46	0.00038	2.353	1.52–3.57	< 0.0001	1.340	0.81–2.17	0.25
**Sociodemographics**									
Sex	1.802	1.46–2.24	< 0.0001	–	–	–		–	–
Age (10 years)	1.884	1.67–2.12	< 0.0001	2.011	1.74–2.34	< 0.0001	1.642	1.34–2.02	< 0.0001
Socioeconomic status	0.979	0.96–1.00	0.092	1.000	0.98–1.03	0.99	0.932	0.89–0.97	0.0019
**Cardiovascular risk**									
Diabetes	1.294	0.99–1.68	0.057	1.405	1.01–2.05	0.015	0.975	0.59–1.55	0.92
Obesity	1.436	1.15–1.78	0.0010	1.448	1.10–1.91	0.0079	1.339	0.94–1.89	0.10
Smoking	1.053	0.77–1.42	0.74	0.998	0.65–1.43	0.91	1.139	0.67–1.85	0.62
Hypertension	1.664	1.30–2.14	< 0.0001	1.289	0.94–1.72	0.13	2.748	1.79–4.34	< 0.0001
Dyslipidemia	2.747	2.24–3.38	< 0.0001	3.287	2.44–4.17	< 0.0001	2.173	1.55–3.04	< 0.0001
FH of MI/Stroke	1.034	0.82–1.30	0.78	0.861	0.58–1.12	0.22	1.363	0.96–1.91	0.076
**Distress**									
Social phobia	1.150	0.69–1.84	0.58	0.754	0.31–1.56	0.46	1.663	0.85–3.02	0.11
Panic	1.634	1.07–2.45	0.020	1.448	0.82–2.67	0.17	1.911	1.03–3.40	0.033
Sleep disturbance	1.038	0.75–1.42	0.82	1.302	0.84–1.93	0.23	0.764	0.45–1.24	0.30

OR, Odds ratio; 95% CI, 95% confidence interval; sex 1 = male, 0 = female; cardiovascular risk and distress: 0 = does not apply, 1 = applies; FH of MI/Stroke, Family History of Myocardial infarction/Stroke; Cardiovascular disease events: Total sample: 443 (out of 9,488); Men: 279 (out of 4,780); Women: 164 (out of 4,708).

As can be seen in Table 2, new onset of anxiousness and chronic anxiousness were both linked to new onset of CVD, together with male sex and higher age, cardiovascular risk factors (obesity, hypertension, dyslipidemia) and panic attacks. Further, sex-specific analyses revealed that chronic anxiousness was only predictive in men in addition to well-known risk factors such as higher age, diabetes, obesity, and dyslipidemia. Chronic anxiousness was not associated with CVD in women. New onset of anxiety was associated with new onset of CVD in women, along with age, low SES, hypertension and dyslipidemia, and panic attacks.

### Chronic anxiousness and mortality

Table 3 presents the associations of different forms of anxiousness and mortality.

**Table 3:** Regression model of different chronological forms of anxiousness, sociodemographic factors, cardiovascular risk factors and distress on all-cause mortality.

Variables	HR	95% CI	p-value	c-index
**Anxiousness**				
previous vs never	1.1886	0.83–1.70	0.35	0.00001
new onset vs never	1.1629	0.75–1.81	0.50	0.00028
chronic vs never	1.2362	0.78–1.97	0.37	0.00008
**Sociodemographics**				
Sex	2.2717	1.70–3.04	< 0.0001	0.01111
Age (10 years)	3.3669	2.80–4.04	< 0.0001	0.11301
Socioeconomic status	0.9862	0.96–1.02	0.37	0.00040
**Cardiovascular risk**				
Diabetes	2.2314	1.67–2.98	< 0.0001	0.00692
Obesity	1.2870	0.98–1.70	0.074	− 0.00005
Smoking	1.9254	1.32–2.80	0.00062	0.00607
Hypertension,	0.7700	0.58–1.03	0.078	0.00101
Dyslipidemia,	1.0014	0.77–1.30	0.99	− 0.00009
FH of MI/Stroke	1.3132	0.99–1.74	0.059	0.00189
**Distress**				
Social phobia	1.4217	0.75–2.70	0.28	0.00217
Panic	1.1768	0.61–2.25	0.62	0.00192
Sleep disturbance	1.2782	0.87–1.88	0.21	0.00325

OR, Odds ratio; 95% CI, 95% confidence interval; sex 1 = male, 0 = female; cardiovascular risk and distress: 0 = does not apply, 1 = applies; FH of MI/Stroke = Family History of Myocardial infarction/Stroke; Death events: 246 (out of 10,566). The C-index is a concordance statistic which considers the censored observation time in time to event data.

As portrayed in Table 3, we observed no association between different chronological forms of anxiousness and mortality considering common risk factors for mortality. The strongest predictors of mortality were age (per 10 years), male sex, diabetes and smoking. For obesity and family history of myocardial infarction/stroke, only a trend for mortality (P < 0.10) was found.

## Discussion

In a large German community sample from middle to old age (40–80 years), about 38% of participants reported at least subclinical levels of anxiousness: 12% of the study population suffered from chronic anxiousness, 10% new onset and nearly 16% previous anxiousness. The focus of this paper was on the potential health effects of chronic anxiousness. Consistent with previous findings from our cohort regarding different kinds of psychological distress^27^, anxious individuals were younger, had a lower socioeconomic status and were more often female. Particularly, the chronically anxious were in an overall poorer mental health condition suffering to a substantial degree from additional social phobia and panic attacks. Anxious individuals were more likely to smoke and to have a family history of stroke and myocardial infarction. New onset of cardiovascular disease and mortality occurred more often in men than in women along with other well-known risk factors, e.g. age and dyslipidemia for cardiovascular disease or smoking for mortality.

As we had expected, chronic anxiousness was related to new onset of CVD. However, sex-specific analyses revealed that this finding was only valid for men. New onset of (but not chronic) anxiousness was associated with new onset of CVD in women only. We suggest different interpretations for our results: Sex-specific results between different types of anxiousness and new onset of CVD might be indicating physiological sex differences in response to acute versus chronic stressors. Previous research on sex-specific immune responses showed an increased susceptibility of women to acute stressors^28^. Overall, men reported more cardiovascular risk factors compared to women (Supplementary Tables S1a, S1b). Yet, chronic anxiousness was associated with new onset of CVD in men even when adjusted for significant cardiovascular risk factors such as diabetes, obesity and dyslipidemia. However, based on our data, we do not know if inflammatory responses may mediate the onset of CVD. Second, it is important to point out that the cross-sectional nature of the association between new onset of anxiousness and new onset of CVD does not allow for causal interpretations. Current anxiousness in women (feeling nervous, worry) may also indicate an emotional reaction to newly diagnosed CVD^29^.

Our study indicated that subclinical chronic anxiousness is not linked to overall mortality. Thus, our findings support previously reported results by Miloyan et al.^16^ who found no increase in overall mortality in anxiety disorders. However, some researchers proposed a relationship between anxiety disorders and all-cause mortality^12–15^. We suggest that the potential interrelation between anxiousness, new onset of CVD and mortality is more complex and deserves attention in future research. Possibly, psychosocial and behavioral factors such as nutrition, smoking or exercising might be mediating variables explaining potential links between anxiety^30,31^ and mortality^32–34^.

Our findings have several implications for future research: Previously contradictory findings on the impact of anxiety on CVD might be due to the fact that the time course of anxiousness (e.g. new onset vs. chronic) was not assessed, and men and women were not differentiated. As recommended by cardiological guidelines^35^, routine assessment, respectively referral for treatment of anxiety disorders is warranted. Assessing the current extent of anxiousness might not be sufficient, we suggest including further questions to inquire its chronicity as well as to assess subclinical symptoms of anxiety disorders. For clinicians we cautiously suggest considering a potential harmful effect of particularly chronic anxiousness in men on CVD.

Strengths refer to the large representative community sample and the comprehensive assessment of multiple sources integrating demographic, psychosocial, and behavioral determinants with mental and somatic comorbidities. Limitations pertain to the fact that the medical diagnoses of cardiovascular disease were self-reported, however, in other publications we have been able to demonstrate good correspondence to functional measures^36^. Participants with less severe complaints are more likely to take part in a community study. This might have led to selection bias towards oversampling those with the less severe anxiety symptoms. Also, we relied on data of validated questionnaires, which has the potential to over- and to underestimate the true prevalence. In our study, we assessed anxiousness via the GAD-2, which is a valid measure for anxiety and anxiousness^37,38^, however, it has only two items covering a period of two weeks. More extensive assessments covering a longer periods of time would be necessary to assess reverse causality and variations of anxiousness symptoms between assessments times. Although our data was taken from three repeated assessments (baseline, 2.5 years, and 5 years later), the interrelations between anxiousness and subsequent CVD need to be explored further in longitudinal study designs.

Further studies should investigate different types and time courses of anxiousness and health behavior to identify underlying variables influencing CVD and mortality^30,31,39^ as well as sex-specific targets for intervention. Due to the limited sample of deceased participants, we were not able to differentiate causes of mortality, nor analyze them separately by sex.

Our research contributes to the current literature as it shows that a) not only diagnosed anxiety disorder and clinically relevant anxiety symptoms but also elevated anxiousness can be harmful and b) that the more chronic such symptoms become, the more harmful they are, at least for men. On the other hand, single episodes of anxiousness earlier in life may not necessarily be harmful to the cardiovascular system. Yet we found that different forms of anxiousness, including chronic anxiousness, were not significantly related to all-cause-mortality, neither for men nor for women.

## Methods

### Study sample

We investigated both, longitudinal and cross-sectional information from 11,643 participants aged between 40 and 80 years enrolled in the Gutenberg Health Study (GHS). Mean age was 59.4 (± 10.8); 49.1% were female. Informed consent was obtained from all participants. Parts of the methods section have been published and described before^40–43^.

Since 2007, the GHS is an ongoing population-based, prospective, observational single-center cohort study in the Rhine-Main region in western Mid-Germany with the primary aim to evaluate and improve cardiovascular risk stratification^44^. Participants were randomly drawn from the local registry of the city of Mainz and the county of Mainz-Bingen. Inclusion criteria were the age between 35 and 75 years. Exclusion criteria were insufficient knowledge of German language and inability to participate due to physical or mental impairment. The sample was stratified 1:1 for gender and residence (rural and urban) in equal strata for decades of age. Out of a sample of 28,533, 52.61% participated (*N* = 15.010; 53.3% of all men and 51.9% of all women). We excluded 5.2% who fulfilled exclusion criteria. Of the non-responders, 22.6% refused participation, and another 20% could not be contacted. A computer-assisted telephone interview (CATI) assessment was conducted 2.5 years later. Participants underwent another full examination after a follow-up period of 5 years (May 2012 to October 2014). The study protocol and sampling design were approved by the local ethics committee (Ethics Committee of the Medical Association of Rhineland-Palatinate) and by the local and federal data safety commissioners. All study investigations have been conducted in line with the Declaration of Helsinki and principles outlined in recommendations for Good Clinical Practice and Good Epidemiological Practice. Accordingly, written informed consent was obtained from all participants prior to entering the study^36^.

### Materials and assessment

Baseline examination in the study center included the evaluation of prevalent classical cardiovascular risk factors and clinical variables, a CATI, and laboratory examinations from venous blood samples, blood pressure, and anthropometric measurements^40^. All examinations were performed according to standard operating procedures by certified medical technical assistants. Baseline examination took about 5 h.

Sociodemographic variables and psychological measures were assessed via self-report: sex, age in years, employment (no/yes), income, living with partner (no/yes), and socioeconomic status (SES). SES was defined according to Lampert and Kroll^45^. The index ranges from 3 (lowest SES) to 21 (highest SES) based on education, profession, and income.

Anxiousness was assessed using the two-item version of the Generalized Anxiety Disorder GAD-7^37,38^, which provides proven convergent validity with established anxiety measures^46^. Two items—‘Feeling nervous, anxious or on edge’ and ‘Not being able to stop or control worrying’ are rated from 0 = ‘not at all’, 1 = ‘several days’, 2 = ‘over half the days’, and 3 = ‘nearly every day’.

Previous research has shown that the a GAD-cut-off ≥ 2 has a higher sensitivity for screening generalized anxiety disorder than a cut-off of 3^47,48^ and a cut-off of 2 can be regarded sufficient for screening for elevated anxiety^49^. Further, self-reported symptoms are never “severe enough” to present a definite diagnosis of generalized anxiety disorder, yet they are relevant for mental and physical health^50,51^. We choose “2” as a cut-off in order to detect clinical as well as subclinical symptoms of anxiety disorders.

Based on self-reported anxiousness consisting of data from three points of time (Baseline; CATI; follow-up), chronic anxiousness was defined as participants beyond cut-off at all three assessments or CATI and follow-up, indicating that the person reported ongoing anxiousness, which has already been present over a period of at least 2.5 years.

The group *never anxious* consisted of persons who were never beyond cut off*. Previous anxiousness* included participants who reported anxiousness beyond cut-off at baseline and/or CATI but not follow-up. *New onset* consisted of subjects who reported anxiousness at follow-up but not previously. Participants who were currently anxious and also had been previously anxious at baseline (N = 617) were excluded from the analyses.

The German version of the three item Mini-Social Phobia Inventory (Mini-Spin) was used to detect *social anxiety*. A cut-off score of 6 (range 0–12) separated individuals with generalized social anxiety disorder and controls with good sensitivity (89%) and specificity (90%)^52^.

*Panic disorder* was screened with the brief Patient Health Questionnaire (PHQ) panic module. Patients were screened positive if at least two of the first four PHQ panic questions were answered with ‘yes’^53^.

*Sleep disturbances* were assessed with the corresponding item of the depression module of the Patient Health Questionnaire (PHQ-9)^54^: “trouble falling or staying asleep, or sleeping too much “ that has to be rated on a 4 point Likert scale ranging from—not at all (= 0), several days (= 1), more than half the days (= 2), nearly every day (= 3.) Clinically relevant sleep disorders were defined by sleep disturbances at least more than half the days over the last 2 weeks^42^.

### Interview assessments

During the CATI, participants were asked whether they had ever received a definite diagnosis of any depressive or anxiety disorder by a physician (medical history of lifetime diagnosis of any depressive, respectively, anxiety disorder)^43^. The presence of coronary heart disease was assessed by the question: ‘Were you diagnosed with a stenosis of your coronary vessels?’ Cardiovascular disease (CVD) was defined if at least one of the following, self-reported disease was present: myocardial infarction, heart failure, stroke, deep vein thrombosis, pulmonary embolism, and peripheral arterial disease; family history of myocardial infarction was assessed by self-report as well^42^.

As cardiovascular risk factors smoking, obesity, unhealthy alcohol intake, diabetes, hypertension, and dyslipidemia were assessed. *Smoking* was dichotomized into nonsmokers (never smoker and ex-smoker) and current smokers (regular and occasional smokers smoking less than 1 cigarette per day^55^). *Obesity* was defined as a Body Mass Index (BMI) of at least 30 kg/m^2^^43^. *Diabetes* was defined in individuals with a definite diagnosis of diabetes by a physician or a blood glucose level of ≥ 126 mg/dl in the baseline examination after an overnight fast of at least 8 h or a blood glucose level of > 200 mg/dl after a fasting period of 8 h. *Hypertension* was defined as a current SBP of at least 140 mmHg or a DBP of at least 90 mmHg, or as intake of antihypertensive medication. *Dyslipidemia* was defined as a definite diagnosis of dyslipidemia by a physician or an LDL/HDL-ratio of > 3.5.

*Mortality* was documented for all mortality incidences with all-cause mortality. Mortality updates were performed by quarterly queries to the registry offices and the mortality registry Rhineland-Palatinate. For death reviews official death certificates were acquired. After follow-up assessment until May 2019, of 11,643 participants, 276 (2.4%) died within a time period of 6.71 years—195 (1.7%) were male and 81 (0.7%) were female.

### Statistical analysis

Absolute numbers, percentages, or means with standard deviations are reported. Comparisons between anxiousness groups (never, previous, new onset, chronic) were analyzed with Kruskal–Wallis tests or Chi^2^-tests. We used complete case analysis, and did not impute missing values. In order to investigate the association between anxiousness and new onset of cardiovascular disease, chronic anxiousness was used as a predictor in multiple generalized linear models with a binominal distribution and a log link function adjusted for sociodemographic variables. Individuals who had reported CVD at baseline were excluded from further analyses. Cox regression (or proportional hazards regression) was used to investigate the influence of chronic anxiousness on mortality. Analyses for new onset of CVD were conducted first for the total sample, then for males and females separately. Due to the low numbers of mortality we did not perform separate analyses by sex.

*P*-values are based on two-tailed tests and are presented as exact values. The level of significance was set at p < 0.05. Research questions were exploratory, thus, no adjustments for multiple testing were performed. We recommend interpreting *p*-values with caution taking effect estimates into account as a large number of tests have been conducted. All statistical analyses were performed using SAS for Windows 9.4 TS Level 1 M1 (SAS Institute Inc.) Cary, NC, USA.

## Supplementary information

Supplementary Tables.
