# Supplementary material for: The association of chronic anxiousness with cardiovascular disease and mortality in the community: results from the Gutenberg Health Study
Source: Sci Rep. 2020 Jul 24;10:12436. doi: 10.1038/s41598-020-69427-8 (PMC7381650; doi:10.1038/s41598-020-69427-8)
Supplement: Supplementary file 1 — Supplementary Tables. [file 41598_2020_69427_MOESM1_ESM.docx]

**The association of chronic anxiousness
with cardiovascular disease and mortality in the community:**

**Results from the Gutenberg Health Study**

Iris Reiner ^a*^, Ana Nanette Tibubos ^a^, Antonia M. Werner ^a^, Mareike Ernst ^a^, Elmar Brähler ^a^, Jörg Wiltink ^a^, Matthias Michal ^a^, Andreas Schulz ^b, i^, Philipp S. Wild ^b, g, h, i^, Thomas Münzel^c,h,i^, Natalie Arnold^c,h,i^, Seyed Hamidreza Mahmoudpour^d^, Karl Lackner^e^, Norbert Pfeiffer^cf^, Manfred E. Beutel ^a^

a Department of Psychosomatic Medicine and Psychotherapy, University Medical Center of the Johannes Gutenberg-University Mainz, Untere Zahlbacher Str. 8, 55131 Mainz, Germany

b Preventive Cardiology and Preventive Medicine - Center for Cardiology, University Medical Center of the Johannes Gutenberg-University Mainz, Langenbeckstr. 1, 55131 Mainz, Germany

c Center for Cardiology - Cardiology I, University Medical Center of the Johannes Gutenberg-University Mainz, Langenbeckstr. 1, 55131 Mainz, Germany

d Institute of Medical Biostatistics, Epidemiology and Informatics, University Medical Center of the Johannes Gutenberg-University Mainz, Obere Zahlbacher Str. 69, 55131 Mainz, Germany

e Institute of Clinical Chemistry and Laboratory Medicine, University Medical Center of the Johannes Gutenberg-University Mainz, Langenbeckstr. 1, 55131 Mainz, Germany

f Department of Ophthalmology, University Medical Center Mainz of the Johannes Gutenberg-University Mainz, Mainz, Germany

g Center for Thrombosis and Hemostasis (CTH), University Medical Center of the Johannes Gutenberg-University Mainz, Langenbeckstr. 1, 55131 Mainz, Germany

h German Center for Cardiovascular Research (DZHK), partner site Rhine-Main, Langenbeckstr. 1, 55131 Mainz, Germany

i Center for Translational Vascular Biology (CTVB), University Medical Center of the Johannes Gutenberg-University Mainz, Langenbeckstr. 1, 55131 Mainz, Germany

*Corresponding author:
PD Dr. Iris Reiner
Department of Psychosomatic Medicine and Psychotherapy, University Medical Center of the Johannes Gutenberg-University
Untere Zahlbacher Str. 8
55131 Mainz, Germany
phone: +49 6131 17- 3511
iris.reiner@unimedizin-mainz.de

**Supplements:**

**Table 1a: Anxiousness in a German community sample: Sociodemographic features, new onset of cardiovascular disease, cardiovascular risk factors and distress – men only.**

**Table 1b: Anxiousness in a German community sample: Sociodemographic features, new onset of cardiovascular disease cardiovascular risk factors and distress – women only.**

**Supplements- S1:**

Table S1a: Anxiousness in a German community sample: Sociodemographic features, new onset of cardiovascular disease, cardiovascular risk factors and distress – men only.

| Anxiousness | **Never** | | **Previous** | **New onset** | **Chronic** | ***p*-value*** |
| --- | --- | --- | --- | --- | --- | --- |
|  | 69%  (n=3922) | | 13.6% (n=774) | 8.7 %  (n=494) | 8.7% (n=498) |  |
| *Sociodemographics* | | | | | | |
| Age in years  (Mean, SD) | | 60.4±10.9 | 58.4±10.6 | 58.4±10.9 | 56.9±9.8 | <0.0001 |
| Socioeconomic status (Mean, SD) | | 13.90±4.39 | 13.76±4.49 | 13.87±4.31 | 13.39±4.27 | 0.098 |
| *Cardiovascular disease  and risk factors (%; n)* | | | | | | |
| New onset CVD | 5.6% (190) | | 5.2% (34) | 6.7% (29) | 9.7% (41) | 0.0026 |
| Diabetes | 12.9 (506) | | 12.3 (95) | 12.0 (59) | 9.4 (47) | 0.036 |
| Obesity | 27 (1060) | | 24.4 (189) | 27.7 (137) | 29.5 (147) | 0.42 |
| Smoking | 14.0 (548) | | 17.3 (134) | 19.3 (95) | 18.9 (94) | <0.0001 |
| Hypertension | 60.1 2349) | | 54.7 (422) | 58.6 (289) | 54.0 (269) | 0.0047 |
| Dyslipidemia | 40.5 (1582) | | 42.3 (327) | 41.4 (204) | 47.9 (238) | 0.0045 |
| FH of MI/Stroke | 20.7 (812) | | 23.1 (179) | 21.7 (107) | 23.7 (118) | 0.090 |
| *Distress (%; n)* | | | | | | |
| Social Phobia | | 0.9 (36) | 2.3 (18) | 5.7 (28) | 20.2 (100) | <0.0001 |
| Panic attack  (past 4 weeks) | | 0.8 (30) | 3.9 (30) | 5.9 (29) | 18.5 (92) | <0.0001 |
| Sleep disturbance | | 4.5 (175) | 8.6 (65) | 12.4 (60) | 20.7 (101) | <0.0001 |

N (men) = 5688

Note: FH of MI/Stroke = Family History of Myocardial infarction/Stroke CVD = Cardiovascular disease cardiovascular risk and distress: 0=does not apply, 1=applies; *chi² or Kruskal Wallis test; numerical values with standard deviation are mean scores.

Table S1b: Anxiousness in a German community sample: Sociodemographic features, new onset of cardiovascular disease cardiovascular risk factors and distress – women only.

| Anxiousness | **Never** | | **Previous** | **New onset** | **Chronic** | ***p*-value*** |
| --- | --- | --- | --- | --- | --- | --- |
|  | 37.8%  (n= 2909) | | 18.2% (n= 967) | 11.5 %  (n= 614) | 15.5% (n= 826) |  |
| *Sociodemographics* | | | | | | |
| Age in years  (Mean, SD) | | 59.9±10.9 | 58.7±10.6 | 58.1±10.5 | 58.0±10.3 | <0.0001 |
| Socioeconomic status (Mean, SD) | | 12.50±4.31 | 12.21±4.20 | 12.86±4.20 | 11.95±4.17 | 0.073 |
| *Cardiovascular disease  and risk factors (%; n)* | | | | | | |
| New onset CVD | 3.1% (83) | | 3.6% (32) | 4.2% (24) | 4.6% (35) | 0.029 |
| Diabetes | 7.7 (224) | | 8.6 (83) | 6.5 (40) | 7.6 (62) | 0.64 |
| Obesity | 24.0 (699) | | 24.7 (239) | 24.4 (150) | 24.5 (202) | 0.76 |
| Smoking | 12.8 (373) | | 14.1 (136) | 15.5 (95) | 17.6 (145) | 0.00031 |
| Hypertension | 49.7 (1443) | | 48.2 (466) | 42.6 (261) | 48.7 (402) | 0.095 |
| Dyslipidemia | 26.0 (753) | | 27.1 (261) | 26.4 (162) | 29.8 (245) | 0.052 |
| FH of MI/Stroke | 23.2 (676) | | 27.9 (270) | 25.1 (154) | 28.5 (235) | 0.0021 |
| *Distress (%; n)* | | | | | | |
| Social Phobia | | 1.9 (54) | 4.3 (41) | 7.6 (46) | 18.8 (154) | <0.0001 |
| Panic attack  (past 4 weeks) | | 1.4 (40) | 5.0 (48) | 8.4 (51) | 22.0 (179) | <0.0001 |
| Sleep disturbance | | 6.6 (187) | 10.9 (103) | 15.3 (93) | 27.1 (221) | <0.0001 |

N (women) = 5316

Note: FH of MI/Stroke = Family History of Myocardial infarction/Stroke CVD = Cardiovascular disease cardiovascular risk and distress: 0=does not apply, 1=applies; *chi² or Kruskal Wallis test; numerical values with standard deviation are mean scores.
